# Cannabinoids affect the mouse visual acuity via the cannabinoid receptor type 2

**DOI:** 10.1038/s41598-020-72553-y

**Published:** 2020-09-25

**Authors:** Bruno Cécyre, Ismaël Bachand, François Papineau, Chloé Brochu, Christian Casanova, Jean-François Bouchard

**Affiliations:** 1 Laboratoire de Neuropharmacologie, École d’optométrie, Université de Montréal, Succursale Centre-Ville, C.P. 6128, Montréal, Québec H3C 3J7 Canada; 2 Laboratoire des Neurosciences de la Vision, École d’optométrie, Université de Montréal, Montréal, Québec Canada

**Keywords:** Visual system, Motion detection, Retina

## Abstract

Recently, there have been increasing indications that the endocannabinoid (eCB) system is involved in vision. Multiple research teams studied the cannabinoid receptor type 2 (CB2R) expression and function in the mouse retina. Here, we examined the consequence of CB2R modulation on visual acuity using genetic and pharmacologic tools. We found that *Cnr2* knockout mice show an enhanced visual acuity, CB2R activation decreased visual acuity while CB2R blockade with the inverse agonist AM630 increased it. The inhibition of 2-arachidonylglycerol (2-AG) synthesis and degradation also greatly increased and decreased visual acuity, respectively. No differences were seen when the cannabinoid receptor type 1 (CB1R) was deleted, blocked or activated implying that CB2R exclusively mediates cannabinoid modulation of the visual acuity. We also investigated the role of cannabinoids in retinal function using electroretinography (ERG). We found that modulating 2-AG levels affected many ERG components, such as the a-wave and oscillatory potentials (OPs), suggesting an impact on cones and amacrine cells. Taken together, these results reveal that CB2R modulates visual acuity and that eCBs such as 2-AG can modulate both visual acuity and retinal sensitivity. Finally, these findings establish that CB2R is present in visual areas and regulates vision-related functions.

## Introduction

In the last years, there have been increasing indications that the endocannabinoid (eCB) system is involved in vision. The cannabinoid receptor type 1 (CB1R) is found in the majority of retinal neurons, including photoreceptors, horizontal cells, bipolar cells, amacrine cells and ganglion cells (for review see^1^). Many studies, based on patch-clamp recordings from retinal slices, found that cannabinoids affected potassium, chloride and calcium currents (for review see^1^). Most of these studies were carried out using compounds such as WIN55,212-2, a synthetic cannabinoid with similar affinity to both CB1R and CB2R. Nowadays, synthetic cannabinoid agonists and inverse agonists with a better selectivity for CB1R or CB2R were developed. Among them, ACEA (CB1R agonist), HU308 (CB2R agonist), AM251 (CB1R inverse agonist) and AM630 (CB2R inverse agonist) were shown to be very selective^2,3^.

There is mounting evidence that CB2R is expressed in neuronal tissues, such as cerebellum, brainstem, hippocampus, cortex, and retina^1^. Indeed, the retinal expression of CB2R was reported in many animals including monkeys^4^, though it remains controversial because of the lack of specificity of antibodies directed against CB2R^5^. However, many studies confirmed the presence of a functional CB2R in the retina with pharmacological and genetic tools. For instance, CB2R was found to be involved in retinal ganglion cell (RGC) axon guidance during development^6^; in vivo recording of electroretinogram (ERG) responses demonstrated that mice lacking CB2R (*Cnr2*^*-/-*^) exhibited an increased a-wave amplitude under scotopic conditions, reflecting an enhanced sensitivity of photoreceptors^7^. Furthermore, a recent report studying the retinal function of *Cnr2*^*-/-*^ mice confirmed that ERG responses are altered in these mice, and found that a prolonged treatment with CB2R inverse agonist AM630 mimics the effects seen in *Cnr2*^*-/-*^ mice^8^. These accumulating facts demonstrate the modulator effect of CB2R, thus suggesting its functional presence in the retina.

Until now, almost all studies regarding CB2R expression and function in visual structures were realized on the retina. Since vision does not rely strictly on the retina, other tests need to be conducted to evaluate the impact of CB2R on the visual function. One of these assays takes advantage of the optomotor response (OMR), consisting of a stereotyped head movement in response to movement in the surrounding environment. This reflex is highly conserved among many species and does not require a training process. Hence, it does not require constrains to animals, which are allowed to move freely on a platform. Compared to ERGs, the OMR can assess deficits in RGCs, optic nerve transmission or brain visual integration.

We investigated the visual acuity and retinal function of mice after genetic manipulation or pharmacological modulation. The results reported here show that transgenic mice lacking CB2R displayed an increased visual acuity. Similarly, the administration of CB2R agonists and inverse agonists respectively decreased and increased visual acuity. Furthermore, the modulation of 2-AG levels affected retinal sensitivity, confirming the functional presence of cannabinoid receptors in the retina and suggesting that eCBs could be implicated in the retinal homeostasis. These results are consistent with the hypothesis that CB2R is expressed in the retina and strengthen the current knowledge of cannabinoids in visual function.

## Results

### The deletion of *Cnr2* enhances the visual acuity during the development through adulthood

**Figure 1:**
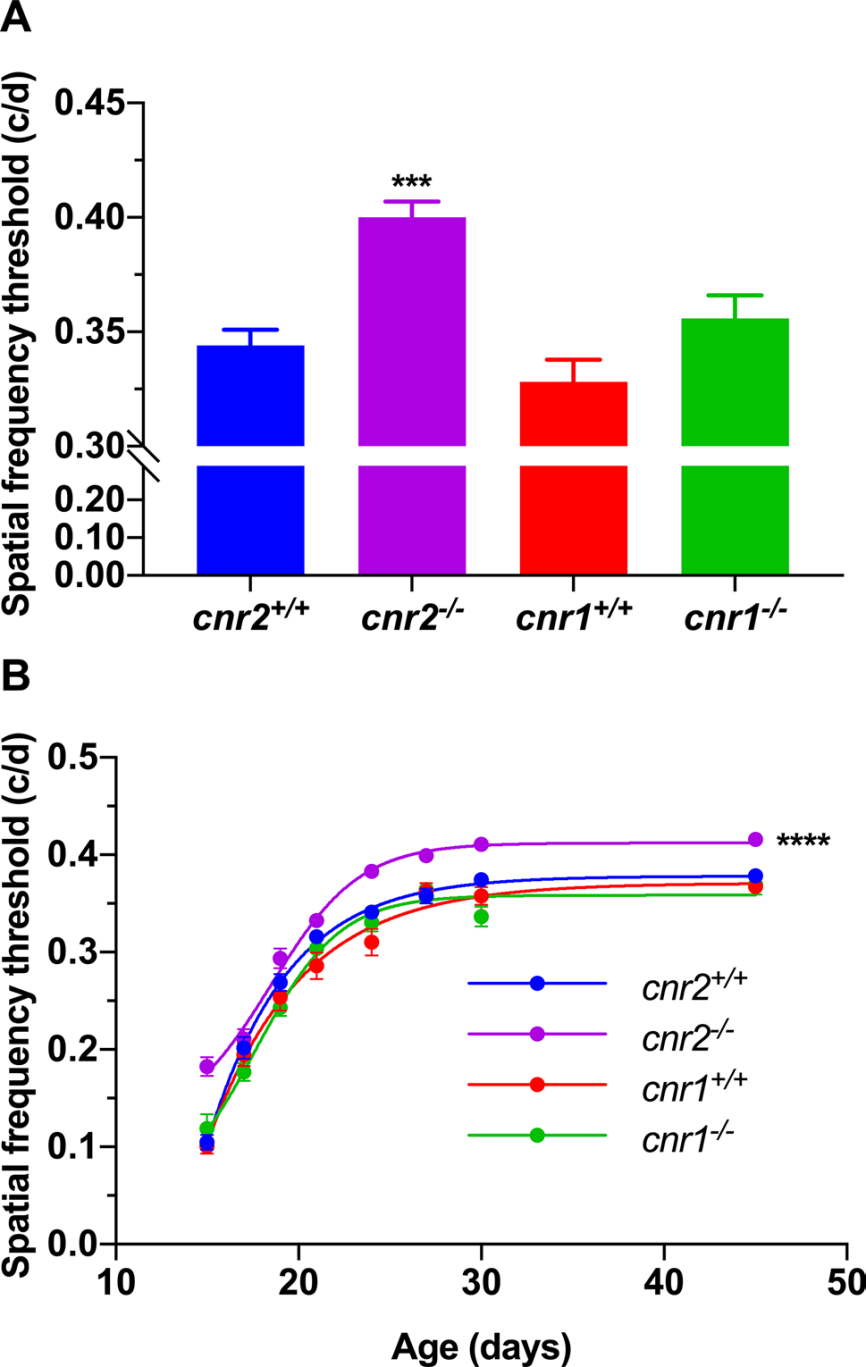
CB2R deletion enhances visual acuity in adults and through postnatal development. **(A,B)** The *Cnr2*^*-/-*^ mice showed a better spatial frequency threshold compared to *Cnr2*^+*/*+^ animals. No differences were observed between *Cnr1*^*-/-*^ and *Cnr1*^+*/*+^ mice. For (**A)** and (**B)** respectively, *Cnr1*^+*/*+^, n = 14 and 38; *Cnr1*^*-/-*^, n = 30 and 38; *Cnr2*^+*/*+^, n = 32 and 37; *Cnr2*^*-/-*^, n = 18 and 32. Statistical analysis was performed by one-way (**A**) or two-way (**B**) ANOVA with Tukey post hoc test. The values are mean ± SEM. *** p = 0.0002, **** p < 0.0001 compared to *Cnr2*^+*/*+^. (c/d: cycles per degree).

Our data indicate that the absence of CB2R enhanced the visual acuity in adults. Indeed, the spatial frequency threshold of *Cnr2*^*-/-*^ mice was significantly higher than the one of *Cnr2*^+*/*+^ mice (Fig. 1A; one-way ANOVA, p = 0.0002). No differences were observed between *Cnr1*^*-/-*^ and *Cnr1*^+*/*+^ mice (Fig. 1A; one-way ANOVA, p = 0.2013).

The important role of cannabinoids during visual system development has been extensively studied in the literature^6,9^. In order to better evaluate the impact of *Cnr2* deletion on visual acuity, we measured the spatial frequency threshold of the same wildtype and mutant mice during their postnatal development, with regular tests between eye opening and adulthood. Similarly to adult animals, the visual acuity of *Cnr2*^*-/-*^ pups was globally enhanced compared to *Cnr2*^+*/*+^ mice (Fig. 1B; two-way ANOVA, p < 0.0001). More specifically, the time points P15, P19, P24, P27, P30 and P45 were statistically different when compared individually between *Cnr2*^*-/-*^ and *Cnr2*^+*/*+^ mice [t-test, p < 0.0001 (P15, P24, P27, P30, P45), p = 0.0164 (P19)]. These results suggest that *Cnr2*^*-/-*^ mice have a better visual acuity from their early development, and they keep this enhanced acuity through adulthood.

### The pharmacological manipulation of CB2R modulates visual acuity

**Figure 2:**
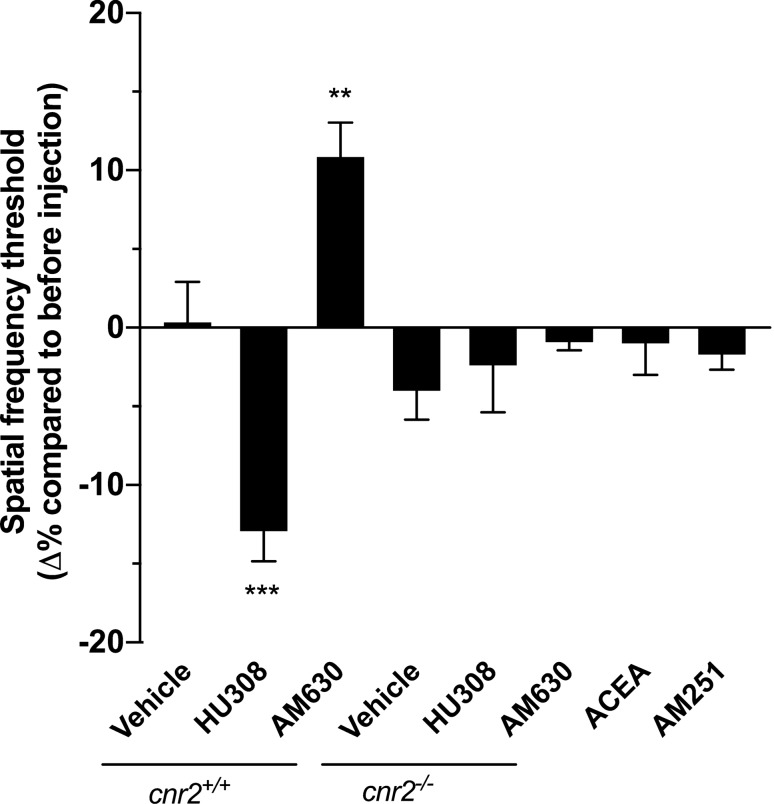
Only CB2R modulates visual acuity. The visual acuity of adult mice was tested, and they received daily i.p. injections of the CB2R agonist HU308 (10 mg/kg), inverse agonist AM630 (2.5 mg/kg), or vehicle for 3 days, after which their visual acuity was tested. The group treated with HU308 showed a decrease in its spatial frequency threshold compared to before injection while the group treated with AM630 had a better visual acuity. Mice lacking CB2R (*Cnr2*^*-/-*^) received the same treatments (HU308 and AM630) and displayed an unchanged visual acuity, demonstrating the specificity of the pharmacological drugs. Adult mice received daily i.p. injections of the CB1R agonist ACEA (2.5 mg/kg), inverse agonist AM251 (3 mg/kg), or vehicle. No changes were seen in their visual acuity. Vehicle, n = 12; HU308, n = 12; AM630, n = 12; vehicle *Cnr2*^*-/-*^, n = 10; HU308 *Cnr2*^*-/-*^, n = 8; AM630 *Cnr2*^*-/-*^, n = 10, ACEA, n = 12, AM251, n = 12. Statistical analysis was performed by one-way ANOVA with Dunnett post hoc test. The values are mean ± SEM. *** p = 0.0007, ** p = 0.0069 compared to the vehicle.

In order to validate the findings obtained with *Cnr2*^*-/-*^ animals, and since compensation could occur in these transgenic animals thus affecting the visual acuity, specific compounds targeting CB2R were injected in adult mice. The CB2R agonist HU308 (10 mg/kg) significantly decreased the visual acuity while the inverse agonist AM630 (2.5 mg/kg) increased it (Fig. 2; one-way ANOVA, p = 0.0007 and p = 0.0069 respectively). The vehicle was tested to make sure that it did not affect the visual acuity. Indeed, no differences were seen after three days of injection. The same drugs were tested on *Cnr2*^*-/-*^ mice in order to validate their specificity (Fig. 2). All compounds, including the vehicle, did not affect the visual acuity in *Cnr2*^*-/-*^ animals, confirming their specificity to CB2R, and also that the dose used for each pharmacological agent did not activate another receptor.

### The CB1R does not modulate visual acuity

Similarly to the CB2R ligands, specific drugs acting on CB1R were injected in adult mice. Both the CB1R agonist ACEA (2.5 mg/kg) and the inverse agonist AM251 (3 mg/kg) did not yield statistically significant changes on visual acuity (Fig. 2; one-way ANOVA). These results suggest that CB1R is not implicated in the cannabinoid modulation of visual acuity.

### Endocannabinoids also modulate visual acuity

**Figure 3:**
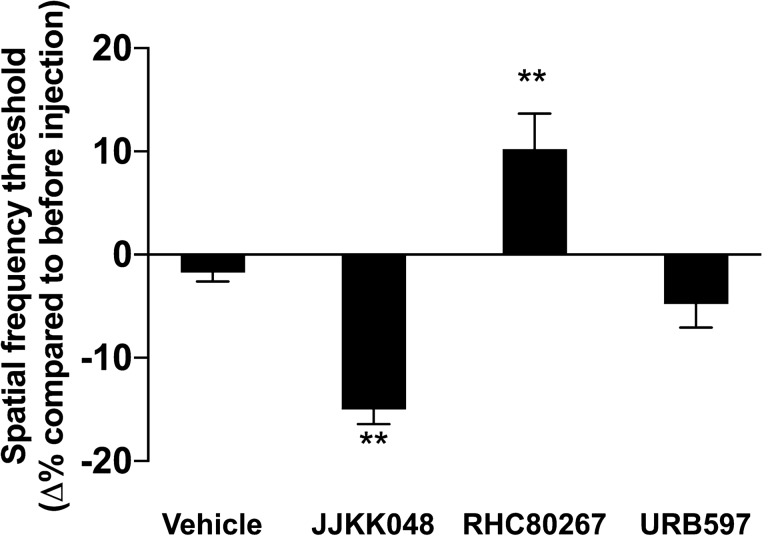
The inhibition of 2-AG degradation decreases visual acuity, while the inhibition of its synthesis increases it. The visual acuity of adult mice was tested, and they received an i.p. injection of MAGL inhibitor JJKK048 (4 mg/kg), DAGL inhibitor RHC80267 (10 mg/kg), FAAH inhibitor URB597 (7.5 mg/kg), or vehicle after which their visual acuity was tested 30 min later. The JJKK048 induced a decrease in the spatial frequency threshold compared to before injection, the RHC80267 provoked an enhancement of the spatial frequency threshold while the URB597 did not reach any statistical significance. Vehicle, n = 12; JJKK048, n = 12; RHC80267, n = 14; URB597, n = 14. Statistical analysis was performed by one-way ANOVA with Dunnett post hoc test. The values are mean ± SEM. ** p = 0.0062 (JJKK048), p = 0.0097 (RHC80267) compared to the vehicle.

So far, we showed that CB2R modulation increased or decreased the visual acuity depending on whether CB2R is blocked or activated, respectively. Since the drugs used are extremely specific, we sought to determine if eCBs could also affect visual acuity. One simple way to increase the levels of eCBs is to block their degradation by specifically targeting the enzyme responsible for it, and vice versa to decrease their levels. In this set of experiments, the inhibitors JJKK048, RHC80267 and URB597, respectively targeting the enzymes MAGL, DAGL and FAAH, were injected 30 min before assessing the visual acuity. By blocking the enzymes MAGL and FAAH, a rapid rise in 2-AG and AEA levels occurs^10,11^, while the inhibition of DAGL causes a decrease in 2-AG levels^12^. The inhibitor JJKK048 (4 mg/kg) strongly decreased visual acuity while the RHC80267 (10 mg/kg) enhanced it (Fig. 3; one-way ANOVA, p = 0.0062 and p = 0.0097 respectively) and the URB597 (7.5 mg/kg) did not significantly affect it.

### The systemic pharmacological manipulation of CB1R and CB2R does not modulate retinal function

**Figure 4:**
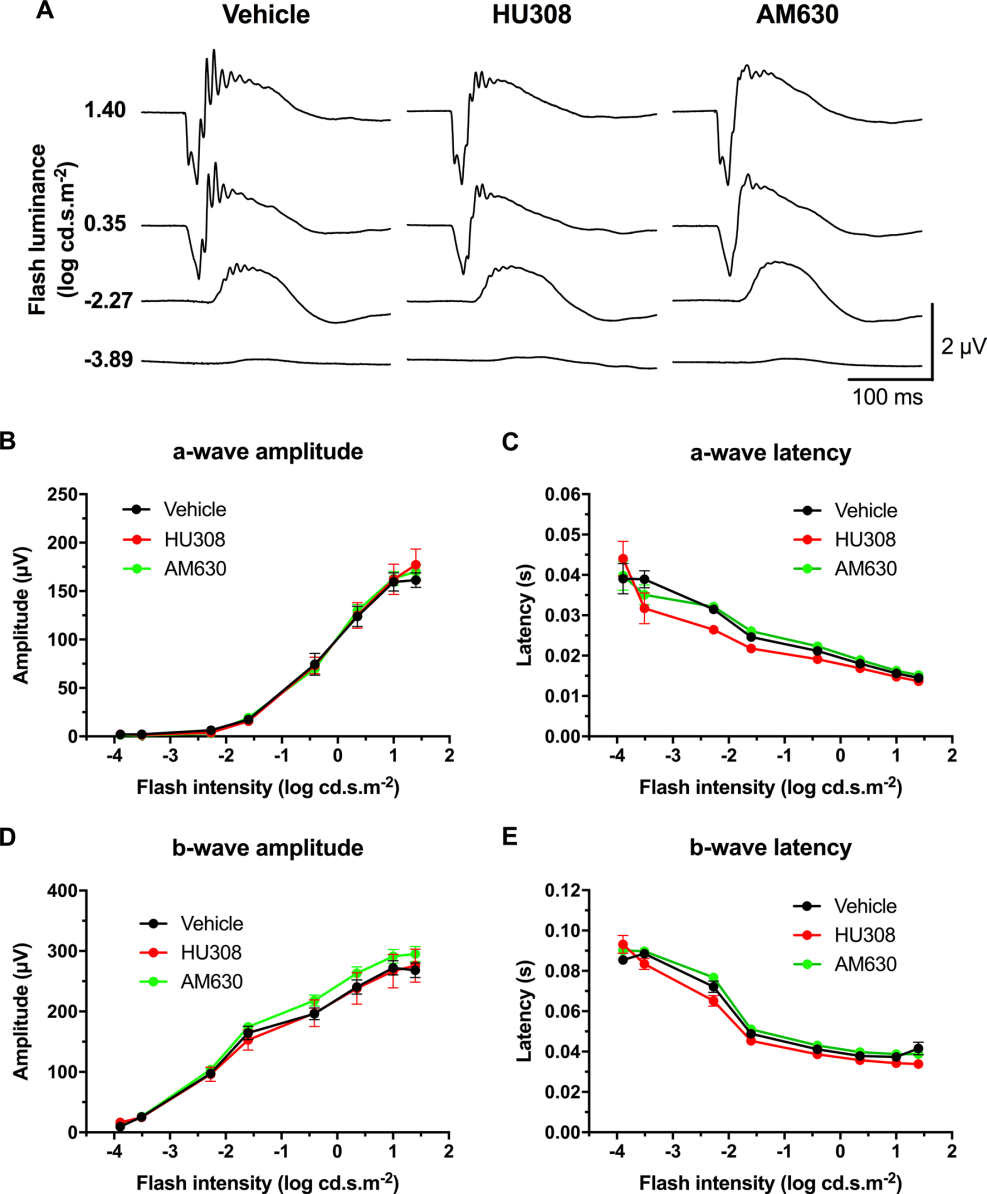
Systemic CB2R modulation does not affect retinal function in scotopic conditions. Adult mice received daily i.p. injections of the CB2R agonist HU308 (10 mg/kg), inverse agonist AM630 (2.5 mg/kg), or vehicle for 4 days, after which their retinal function was tested. (**A**) Mean scotopic ERG traces with the different treatments. The luminance-response function of each animal was established by presenting progressively brighter flashes (bottom to top). Amplitude, time to peak of scotopic ERG a- (**B,C**) and b-waves (**D,E**) respectively, plotted as a function of flash luminance. Vehicle, n = 5; HU308, n = 6; AM630, n = 6. Statistical analysis was performed by two-way ANOVA. The values are mean ± SEM from all animals in each group.

**Figure 5:**
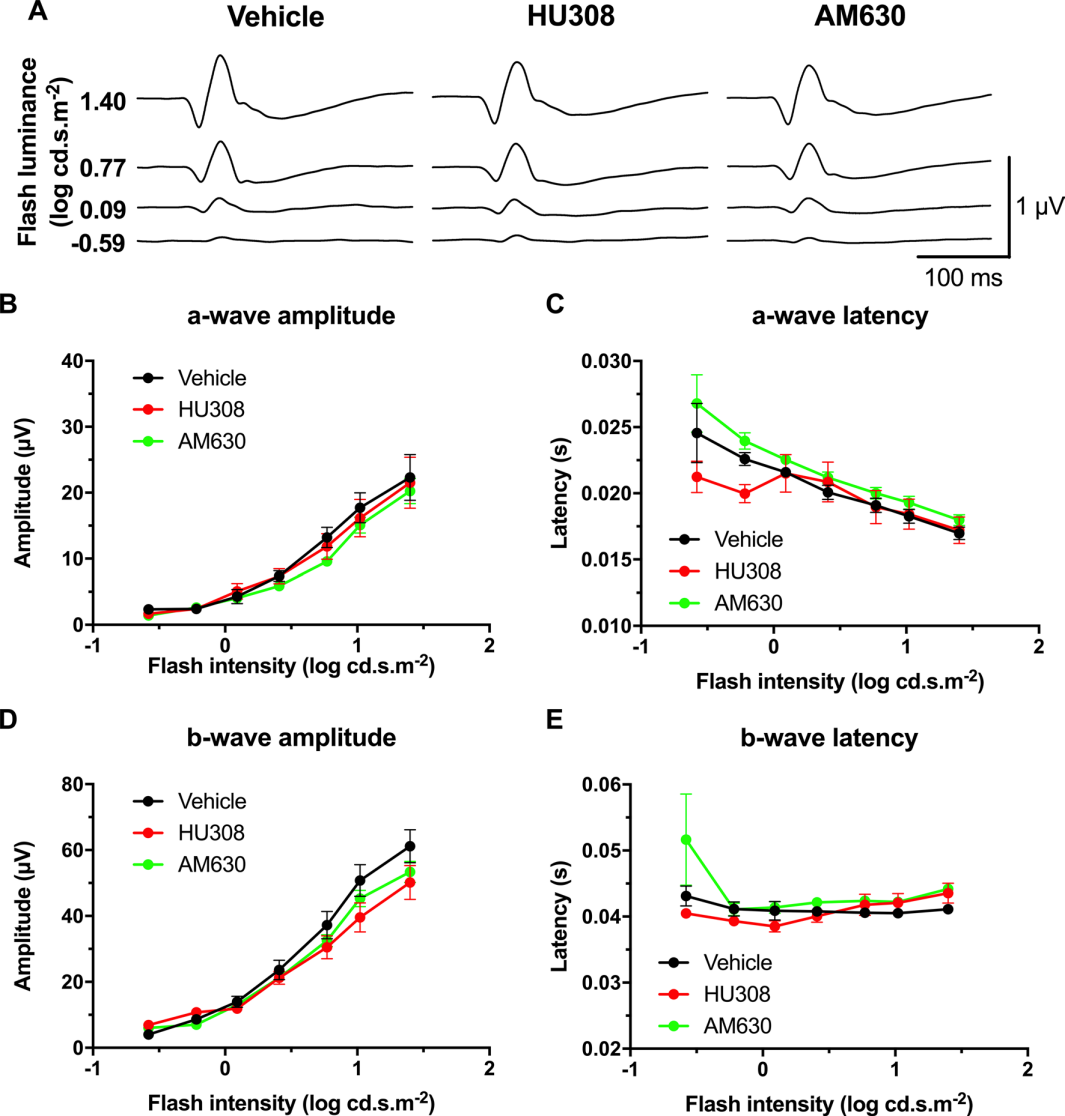
Systemic CB2R modulation does not affect retinal function in photopic conditions. **(A**) Mean photopic ERG traces with the different treatments. The animals were adapted to light for 20 min, and then the luminance-response function of each animal was established by presenting progressively brighter flashes (bottom to top). Amplitude, time to peak of photopic ERG a- (**B,C**) and b-waves (**D,E**) respectively, plotted as a function of flash luminance. Vehicle, n = 5; HU308 (10 mg/kg), n = 6; AM630 (2.5 mg/kg), n = 6. Statistical analysis was performed by two-way ANOVA. The values are mean ± SEM from all animals in each group.

In order to determine whether the enhanced visual acuity found after the administration of cannabinoids came from an increase in retinal sensitivity, ERGs were recorded following OMR testing. Mean scotopic ERG traces of CB2R modulation are shown in Fig. 4A. Both CB2R agonist and inverse agonist, HU308 (10 mg/kg) and AM630 (2.5 mg/kg) respectively, did not significantly alter the a- and b-wave amplitude and time to peak in scotopic conditions (Fig. 4B–E; two-way ANOVA). They also did not affect the amplitude and time to peak of OPs (Supplementary Fig. 1–2; two-way ANOVA). Mean photopic traces of CB2R modulation are presented in Fig. 5A. Both HU308 (10 mg/kg) and AM630 (2.5 mg/kg) did not affect the a- and b-wave amplitude and time to peak in photopic conditions (Fig. 5B–E; two-way ANOVA). CB1R modulation did not modify the retinal function either as both CB1R agonist ACEA (2.5 mg/kg) and inverse agonist AM251 (3 mg/kg) did not induce any change in a- and b-wave amplitude and time to peak in scotopic conditions (Supplementary Fig. 3; two-way ANOVA). They also did not affect the amplitude and peak time of OPs (Supplementary Fig. 4–5; two-way ANOVA). The same outcome was observed in photopic conditions, as ACEA (2.5 mg/kg) and AM251 (3 mg/kg) did not affect the a- and b-wave amplitude and time to peak (Supplementary Fig. 6; two-way ANOVA).

**Figure 6:**
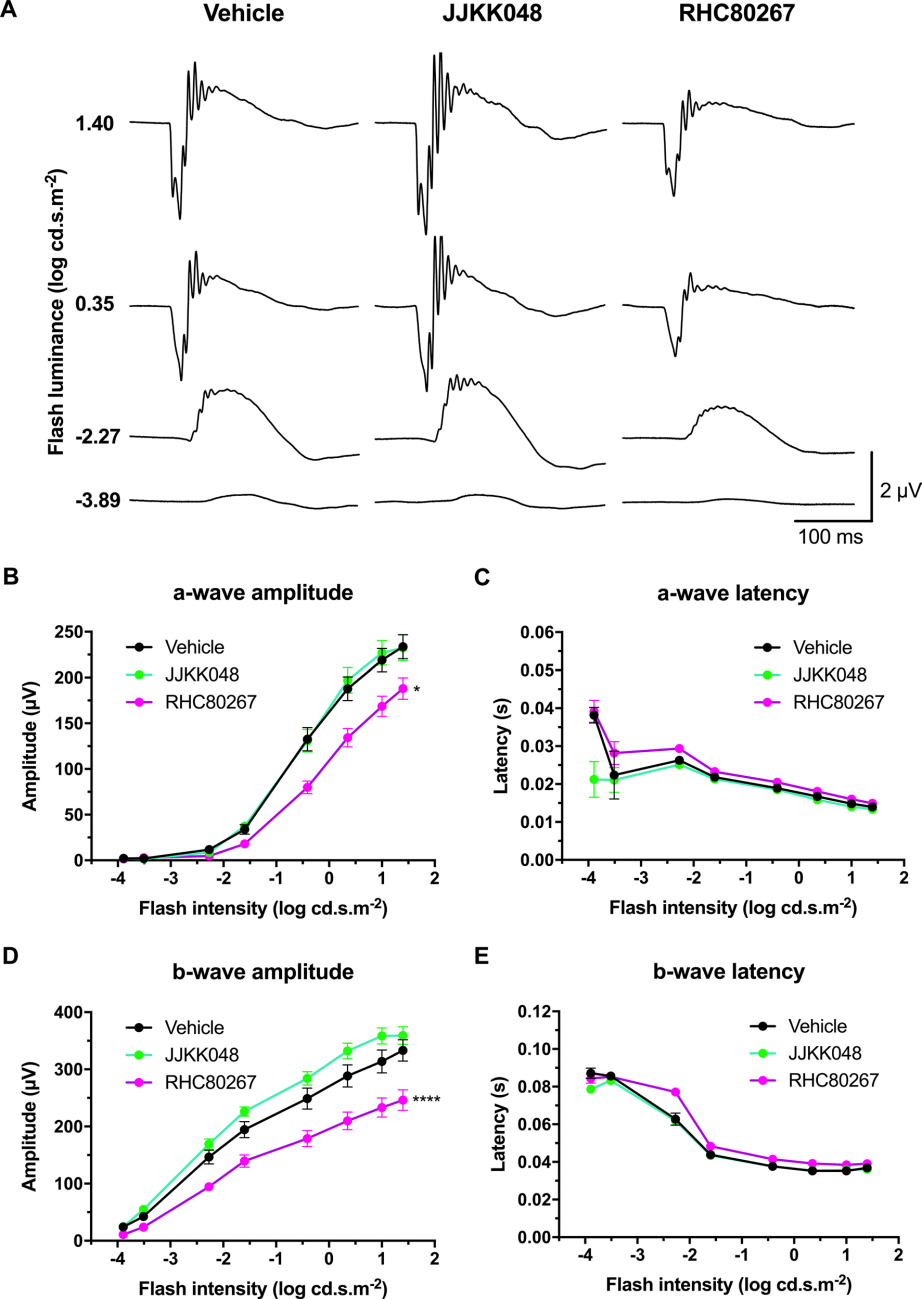
Endocannabinoid modulation affects retinal function in scotopic conditions. Adult mice received an i.p. injection of the MAGL inhibitor JJKK048 (4 mg/kg), DAGL inhibitor RHC80267 (10 mg/kg), or vehicle. Thirty minutes after the injection, their retinal function was tested. A second dose was administered 60 min after the first to act as a booster. (**A**) Mean scotopic ERG traces with the different treatments. The luminance-response function of each animal was established by presenting progressively brighter flashes (bottom to top). Amplitude, time to peak of scotopic ERG a- (**B,C**) and b-waves (**D,E**) respectively, plotted as a function of flash luminance. Vehicle, n = 7; JJKK048, n = 10; RHC80267, n = 7. Statistical analysis was performed by two-way ANOVA with Dunnett post-hoc test. The values are mean ± SEM from all animals in each group. **** p < 0.0001, * p = 0.0106 compared to the vehicle.

### Endocannabinoid modulation affects retinal function

**Figure 7:**
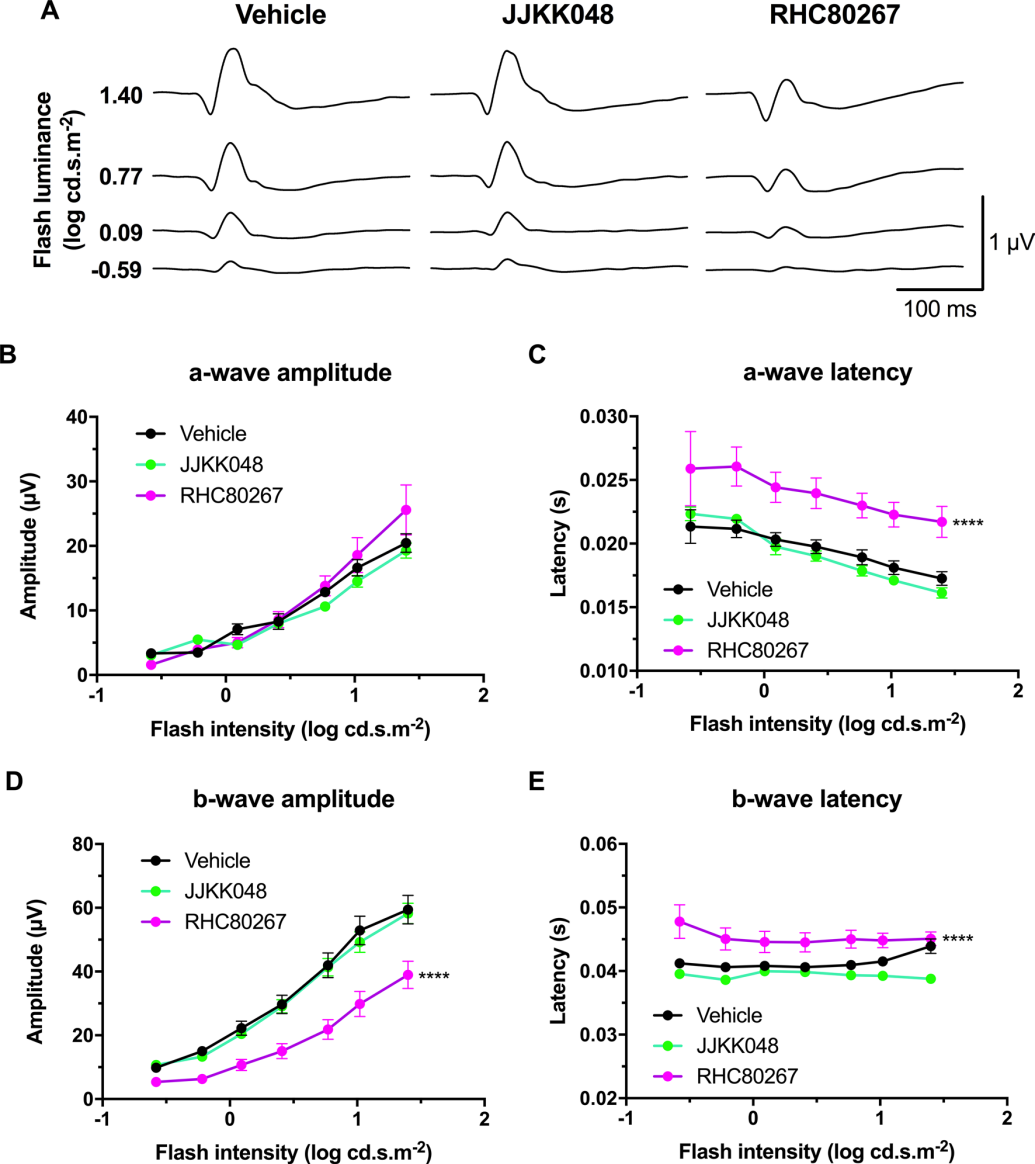
Endocannabinoid modulation affects retinal function in photopic conditions. **(A**) Mean photopic ERG traces with the different treatments. The animals were adapted to light for 20 min, and then the luminance-response function of each animal was established by presenting progressively brighter flashes (bottom to top). Amplitude, time to peak of photopic ERG a- (**B,C**) and b-waves (**D,E**) respectively, plotted as a function of flash luminance. Vehicle, n = 7; JJKK048 (4 mg/kg), n = 10; RHC80267 (10 mg/kg), n = 7. Statistical analysis was performed by two-way ANOVA with Dunnett post-hoc test. The values are mean ± SEM from all animals in each group. **** p < 0.0001 compared to the vehicle.

**Figure 8:**
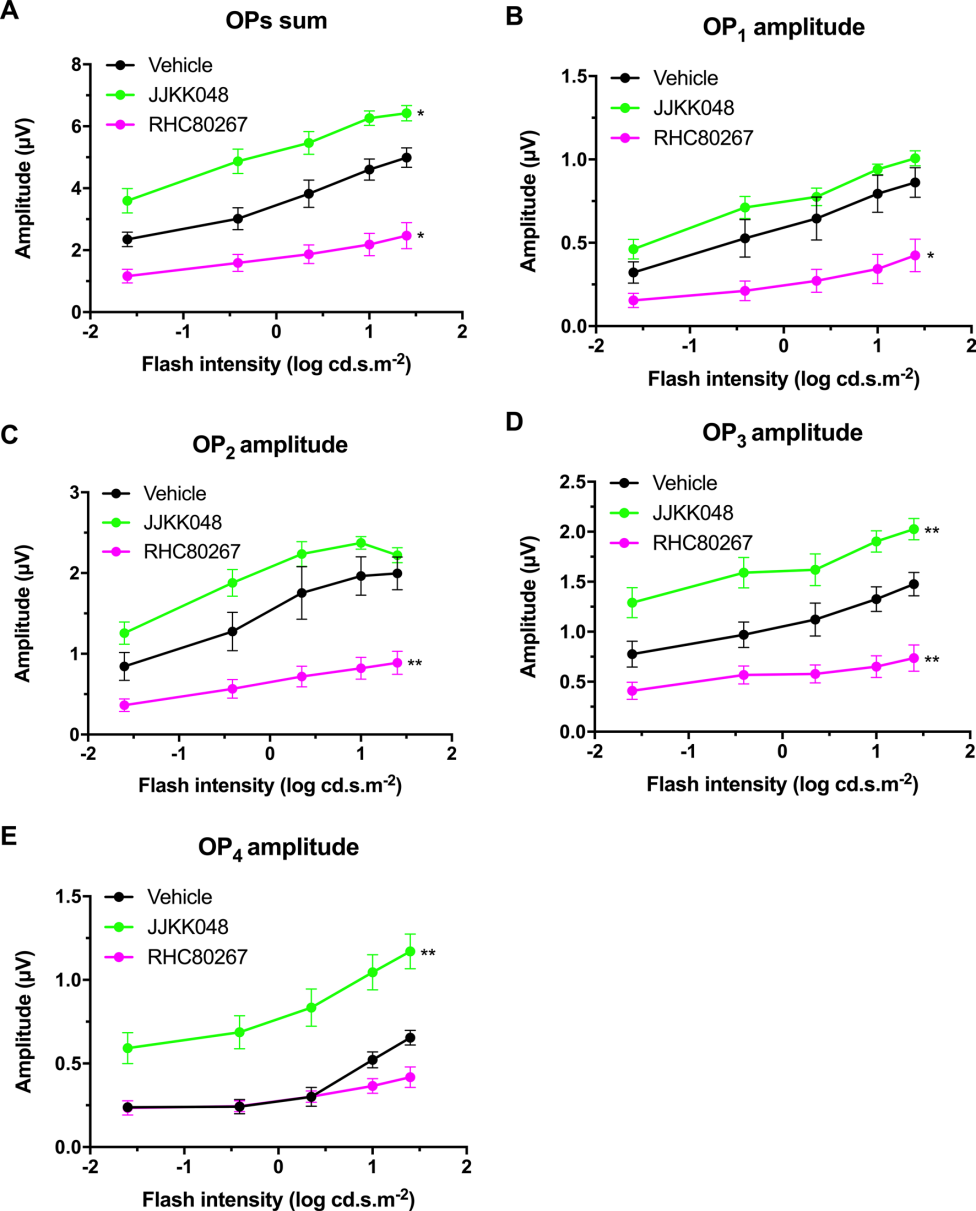
Endocannabinoid modulation affects the amplitude of oscillatory potentials in scotopic conditions. Amplitude of the sum of all oscillatory potentials (**A**) and individual oscillatory potentials (OP_1_–OP_4_) (**B–E**), plotted as a function of flash luminance. Vehicle, n = 7; JJKK048 (4 mg/kg), n = 10; RHC80267 (10 mg/kg), n = 7. Statistical analysis was performed by two-way ANOVA with Dunnett post-hoc test. The values are mean ± SEM from all animals in each group. ** p = 0.0083 (**C**, RHC80267), p = 0.0067 (**D**, JJKK048), p = 0.0073 (**D**, RHC80267), p = 0.0027 (**E**, JJKK048), * p = 0.0439 (**A**, JJKK048), p = 0.0162 (**A**, RHC80267), p = 0.0216 (**B**, RHC80267) compared to the vehicle.

The impact of 2-AG on retinal function was also evaluated. The inhibitor RHC80267 (10 mg/kg), specific for DAGL and thus reducing 2-AG levels, decreased the a- and b-wave amplitude in scotopic conditions (Fig. 6A,B,D; two-way ANOVA, p = 0.0106 and p < 0.0001). Neither RHC80267 nor JJKK048 treatment affected the a- and b-wave latency (Fig. 6C,E; two-way ANOVA) in scotopic conditions. In photopic conditions, the RHC80267 increased both a- and b-wave latency (Fig. 7C,E; two-way ANOVA, p < 0.0001) and decreased b-wave amplitude (Fig. 7D; two-way ANOVA, p < 0.0001). The inhibition of 2-AG degradation by the inhibitor JJKK048 also increased the amplitude of most individual OPs and the sum of all OPs in scotopic conditions (Fig. 8A–E; two-way ANOVA, p-value ranging from 0.0439 to 0.0027), but did not affect their time to peak (Supplementary Fig. 7; two-way ANOVA). Similarly, the inhibitor RHC80267 decreased the amplitude of most individual OPs and the sum of all OPs in scotopic conditions (Fig. 8A–E; two-way ANOVA, p-value ranging from 0.0216 to 0.0073), but did not modify their peak time (Supplementary Fig. 7; two-way ANOVA).

## Discussion

We have tested the impact of genetic and pharmacological modulation of CB2R on the visual acuity and the retinal sensitivity of mice. We report that deletion or the blockade of CB2R both increased the visual acuity, while its activation decreased it. On the other hand, only the modulation of eCB levels affects ERG responses.

### The CB2R is implicated in visual acuity

Earlier reports suggest that acute effects on vision from smoking cannabis include a reduction in Vernier and Snellen acuities, alterations in color discrimination and increases in photosensitivity^13,14^. Anecdotal reports also show that some Jamaican fishermen smoke cannabis to improve dim light vision when fishing at night^15^. These results are corroborated by another study that measured precisely the night vision of Moroccan fishermen using cannabis to improve visual perception^16^. Our results are in line with the previous literature as we precisely demonstrated the impact of cannabinoids on visual acuity.

Recent reports show that CB2R deletion and blockade increase the a-wave amplitude in ERG experiments, reflecting an increased photosensitivity^7,8^. In this study, we found that deletion or blockade of CB2R both increased the visual acuity, while the activation of CB2R decreased it. Since the OMR is a test examining vision as a whole, our results represent a great step forward in our understanding of the impact of cannabinoids on vision.

### The CB1R does not modulate visual acuity and retinal sensitivity

A few years ago, we demonstrated that *Cnr1* deletion had no impact on retinal responses^7^. It was also the case here since *Cnr1* deletion, blockade or activation did not induce a change in visual acuity. This suggests that only CB2R is responsible for the visual effects of cannabinoids.

### Endocannabinoids can act on visual acuity

In many reports studying the impact of cannabinoids in vivo, the doses used did not necessarily represent physiological conditions. Under physiological conditions, eCBs are produced and released on demand. In a set of experiments, we showed that inhibition of MAGL, which degrades 2-AG, decreased visual acuity, indicating 2-AG’s implication in visual functions. Similarly, we observed that the inhibition of DAGL, the enzyme responsible for the synthesis of 2-AG, increased visual acuity. We also found that URB597, which inhibits the enzyme FAAH, responsible for the degradation of AEA, did not affect visual acuity. This suggests that 2-AG is responsible for at least some of the physiological effects of endocannabinoids on visual acuity.

### The CB2R is not implicated in retinal sensitivity

Given that *Cnr2* gene deletion affects ERG responses and that visual acuity is altered by CB2R ligands, it is surprising to observe no effect of the pharmacological CB2R modulation on the retinal sensitivity^7,8^. Many hypotheses could explain this situation. First, this discrepancy could originate from a compensation in *Cnr2*^*-/-*^ animals that does not reflect the true role of CB2R in the retina. A second explanation relates to possible pharmacokinetic issues such as a poor retinal distribution of the compounds or a fast metabolism of the administered drugs. Further research is necessary to elucidate this question.

It should be noted that the impact on visual acuity of injected cannabinoids was observed after a daily injection for three consecutive days, whereas a similar study on the ERG tested the CB2R inverse agonist AM630 injected twice daily for 7 days^8^. The authors of this study reported that since a single treatment with the same drug did not modify the ERG responses, they had to block CB2R for a prolonged time in order to obtain an effect on ERG, claiming that it mimicked adaptive or developmental effect of *Cnr2* deletion. In fact, it seems much more plausible that, in their study, the acute injection of AM630 did not yield an effect because of a fast rate of body distribution. Indeed, plasmatic levels of cannabinoids rise very quickly after administration, usually in 3 to 10 min^17^. It is thus plausible that the effect of an acute injection of cannabinoids cannot be observed if the ERG recordings are not accomplished just after the injection.

If acute injections did not affect ERG responses, why chronic injections did? With chronic use, cannabinoids could potentially accumulate in adipose tissues. The subsequent release of cannabinoids may result in the persistence of cannabinoid activity for several days post administration^18^. A longer elimination half-life is observed in heavy cannabis users, attributable to a slow redistribution from deep compartments such as fatty tissues^19^. Thus, when cannabinoids are administered chronically, the body fat acts as a reservoir and redistributes them over a longer period of time. This would explain why only chronic injections of cannabinoids affect ERG responses.

Furthermore, the effects reported by Borowska-Fielding et al. after a prolonged treatment with CB2R inverse agonist differ from other data presented in the same report^8^. For instance, in their study, the authors found that the a-wave amplitude was nearly identical between acute and chronic AM630 treatments (about 155 µV). However, the a-wave amplitude of the vehicle group was dramatically lower in the chronic treatment (acute: 155 µV; chronic: 95 µV). Thereby, the AM630 yielded the same a-wave amplitude between acute and prolonged treatments while the a-wave amplitude of the vehicle changed drastically. The same phenomenon is observed for the b-wave as well, making overall interpretation of these results rather difficult.

The vehicle can also dramatically change the bioavailability of drugs, especially lipophilic compounds such as cannabinoids. Unfortunately, the vehicle composition was not specified in that same study^8^. It is likely that the vehicle was composed of a certain amount of dimethyl sulfoxide (DMSO). However, this solvent is known to cause toxicity, visible on ERG recordings^20^. In our experiments, great care was taken to dilute the compounds with the least possible amount of DMSO, reaching a 5% level. It is possible that, in Borowska-Fielding et al., that the concentration of DMSO was higher and induced retinal toxicity since the ERG responses were altered in the vehicle group of the prolonged treatment. However, since the AM630 treatment yielded normal ERG responses, it may also be possible that CB2R blockade protected the retina from DMSO-induced retinal toxicity. This interesting hypothesis needs to be deepened.

Many of the first reports studying the impact of cannabinoids on retinal slices used ligands such as WIN55,212-2, a synthetic cannabinoid with similar affinity to both CB1R and CB2R^21,22^. Although these pioneer reports revealed a role for cannabinoids in retinal processing, they could not precisely describe the mechanism by which cannabinoids affect retinal function. Over time, ligands with a stronger affinity for CB1R or CB2R were developed, and they now represent reliable tools to study CB1R and CB2R signaling^2,23^.

### Endocannabinoids can act on retinal function

In ERG experiments, both the inhibition of MAGL and DAGL by JJKK048 and RHC80267 respectively had a major impact. For instance, both treatments had a strong effect on the amplitude of OPs, implying that 2-AG affects amacrine cells and thus further suggesting that cannabinoids modulate retinal functions in a dopamine-dependent mechanism^24,25^. The impact of RHC80267 on the photopic b-wave amplitude and latency suggests that 2-AG affects cones rather than rods. Thus, the inhibition of 2-AG synthesis decreased the sensitivity of cones and caused a delay in their response.

It could be surprising to observe that exogenous cannabinoids did not affect ERG responses while the inhibition of 2-AG synthesis or degradation did. An explanation could be that, since the chemical structure of synthetic cannabinoids and 2-AG inhibitors is different, their body distribution could also be very different. It is thus possible that these compounds were able to reach the retina and affect ERG responses. These results are promising since they highlight a role for 2-AG in retinal function. They are coherent with a report showing the impact of DAGL deletion on contrast and spatial frequency sensitivity^26^. Hence, it would be interesting to test transgenic animals for DAGL and MAGL proteins in ERG to confirm the results obtained with specific inhibitors of 2-AG synthesis and degradation.

In conclusion, this study confirms the major impact of cannabinoids on visual acuity. Most importantly, it demonstrates that the effects observed on visual processing are modulated by CB2R, and not CB1R, and strengthens the importance of CB2R in the mechanisms subtending vision.

## Methods

### Animals

All procedures were performed in accordance with the guidelines from the Canadian Council on Animal Care and the ARVO Statement for the Use of Animals in Ophthalmic and Vision Research. They were approved by the Ethics Committee on animal research of the Université de Montréal. The *Cnr1*^*-/-*^ and *Cnr2*^*-/-*^ transgenic mice were obtained from Beat Lutz (Institute of Physiological Chemistry and Pathobiochemistry, University of Mainz, Germany) and Jackson Laboratory (Bar Harbor, ME, USA), respectively. The *Cnr1*^*-/-*^ and *Cnr2*^*-/-*^ mice were on a C57BL/6N and C57BL/6J genetic background, respectively. Both transgenic mice were compared to background and age-matched WT controls from separate colonies. The colonies were maintained in-house, under a 12 h dark/light cycle. For experiments using adult animals, male and female adult mice aged between 3 and 4 months old were used. For experiments during postnatal development, littermates had one of their toes tattooed at 10 days with a tattoo paste (Ketchum, Brockville, ON, Canada) for identification. Their visual acuity was tested at different times, namely at postnatal (P) days 15, 17, 19, 21, 24, 27, 30 and 45.

### Optomotor response measurements

The visual acuity was determined by using the OptoMotry optokinetic system (CerebralMechanics, Lethbridge, AB, Canada). Briefly, an animal was placed on a platform where stimuli with patterns of varying spatial frequencies were projected. A 1-up-1-down staircase testing protocol was used until head tracking was no longer detected, thereby establishing the visual acuity. Hence, the visual acuity consists of the highest spatial frequency perceivable with 100% contrast. By changing the direction of the stimulus pattern, both eyes can be tested individually. Therefore, thresholds determined from individual eyes were considered individually for statistical analyses. All experiments were carried out at light opening (8 a.m.) by an experienced observer, in blind conditions. Since many mice would get agitated when they came to the testing platform for the first time, they were acclimatized by being placed on the OMR platform for about 10 min the day before the OMR test.

### Electroretinography

After an overnight dark adaptation (at least 12 h of complete darkness), mice were anaesthetized using isoflurane inhalation. The corneas were anesthetized with a drop of 0.5% proparacaine hydrochloride and pupils were dilated with a drop of 1% tropicamide. The mice were then positioned on a probed heating pad and located in a Ganzfeld dome that housed a photostimulator. The ERGs were recorded with a gold electrode inserted in a corneal lens adapted for mice (LKC Technologies, Gaithersburg, MD). Reference and ground electrodes (E2 subdermal electrode; Grass Instruments) were inserted subcutaneously in the forehead and in the tail. Broadband ERGs and oscillatory potentials (OPs) were recorded simultaneously (bandwidth, 1–1000 Hz; 10,000x; P511, Grass, West Warwick, RI). Signals were fed to an analog–digital interface (1401, CED, Cambridge, UK) and were acquired using the software Signal (v.3.01x, CED, Cambridge UK). Scotopic luminance-response functions were obtained in response to progressively brighter stimuli spanning a 5.29 log-unit range (interstimulus interval: 30 s; averaged over 5 flashes; luminance intervals − 3.89 to 1.40 log scot.cd.s.m^−2^). The photopic (cone-mediated) signal was recorded thereafter: the animal was adapted to light background of 30 cd.m^−2^ for 20 min, then ERGs were recorded using a photopic luminance-response function (flash luminance intervals: − 0.59 to 1.40 log photo.cd.s.m^−2^; interstimulus interval: 3 s, averaged over 15 flashes). All experiments were carried out by an experienced observer, in blind conditions.

### Electroretinography analysis

Analysis of the ERG waveforms was performed according to standard practice, but using a 60 Hz low pass digital filter to eliminate the contaminating noise from OPs when analyzing the a- and b-waves^27^. The amplitude of the a-wave was measured from the baseline to the most negative trough, whereas the b-wave amplitude was measured from the trough of the a-wave to the highest positive peak of the retinal response. Implicit times were measured from flash onset to the peak of the waves. OPs were also analyzed similarly using a 60 Hz high pass digital filter to eliminate the a- and b-waves. The amplitude of OPs was measured and reported individually and as the sum of all OPs. Both eyes were recorded independently, but were averaged for each animal. All analyses were carried out by an experienced observer blind to experimental conditions.

### Drug injections

HU308, JJKK048, URB597, RHC80267 and ACEA were purchased from Bio-Techne Canada (Oakville, ON, Canada). AM630 and AM251 were purchased from Cayman Chemical (Ann Arbor, MI, USA). HU308, AM630, and AM251 were first dissolved in dimethyl sulfoxide (DMSO; Fisher Scientific, Ottawa, ON, Canada), while JJKK048 and URB597 were first dissolved in polyethylene glycol 400 (PEG 400; Fisher Scientific). All compounds were then diluted with saline containing Tween 80 (Fisher Scientific), so that the final dilution was composed of 5% DMSO (or 5% PEG 400) and 5% Tween 80. In some cases, sonication and gentle heating were required to obtain a soluble dilution. All compounds were freshly diluted and sterile filtered before injection.

The dose administered for each compound was based on previous work in the literature. The doses are the following: HU308, 10 mg/kg^28,29^; AM630, 2.5 mg/kg^30^; JJKK048, 4 mg/kg^31^; AM251, 3 mg/kg^32^; ACEA, 2.5 mg/kg^33^; URB597, 7.5 mg/kg^34^; RHC80267, 10 mg/kg. All compounds were injected intra-peritoneally (i.p.) and the injection volume was of 6 ml/kg.

Since cannabinoids are poorly soluble and their body distribution is not reliable, animals received one injection daily for 4 days. As ERG recordings require the animals to be dark adapted, visual acuity and ERG tests could not be conducted on the same day. The animals were first tested for baseline visual acuity (day 0, at 8 a.m.), received a daily injection of cannabinoids (days 1–3, at 5 p.m.), tested for visual acuity (day 4, at 8 a.m.), received an injection of cannabinoids (day 4, at 5 p.m.), were dark adapted for 12 h, and tested for ERG recordings (day 5). They had their visual acuity tested on the fourth day and were tested for electroretinography on the fifth day.

Since the compounds JJKK048, URB597 and RHC80267 act rapidly and affect drastically 2-AG and AEA levels^10,11^, they were injected once, 30 min before testing the visual acuity. In electroretinography experiments, a booster dose was administered 60 min after the first dose and consisted of half the initial dose.

### Group sizes and statistics

The group sizes were determined using the resource equation method (see^35^ for review). Statistical analyses were performed using one-way ANOVAs or two-way ANOVAs with Tukey or Dunnett post-hoc test (SPSS 20, IBM, Somers, NY, USA).

## Supplementary information

Supplementary Information.
