# Supplementary material for: Cannabinoids affect the mouse visual acuity via the cannabinoid receptor type 2
Source: Sci Rep. 2020 Sep 25;10:15819. doi: 10.1038/s41598-020-72553-y (PMC7519129; doi:10.1038/s41598-020-72553-y)

Supplementary Information

Cannabinoids Affect the Mouse Visual Acuity via the Cannabinoid Receptor Type 2

Bruno Cécyre, Ismaël Bachand, François Papineau, Chloé Brochu, Christian Casanova, Jean-François Bouchard

Supplementary Figure 1

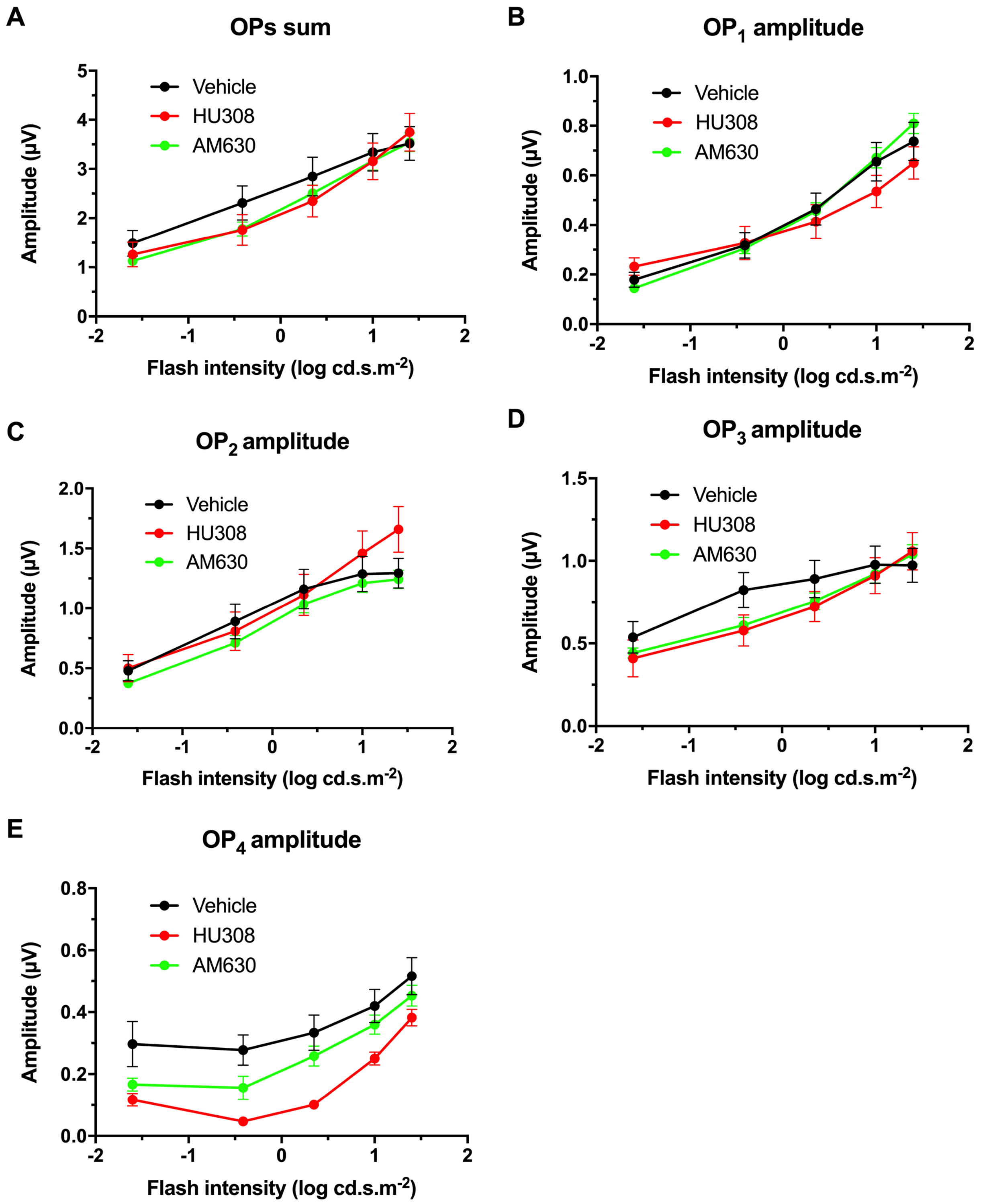

Supplementary Figure 2

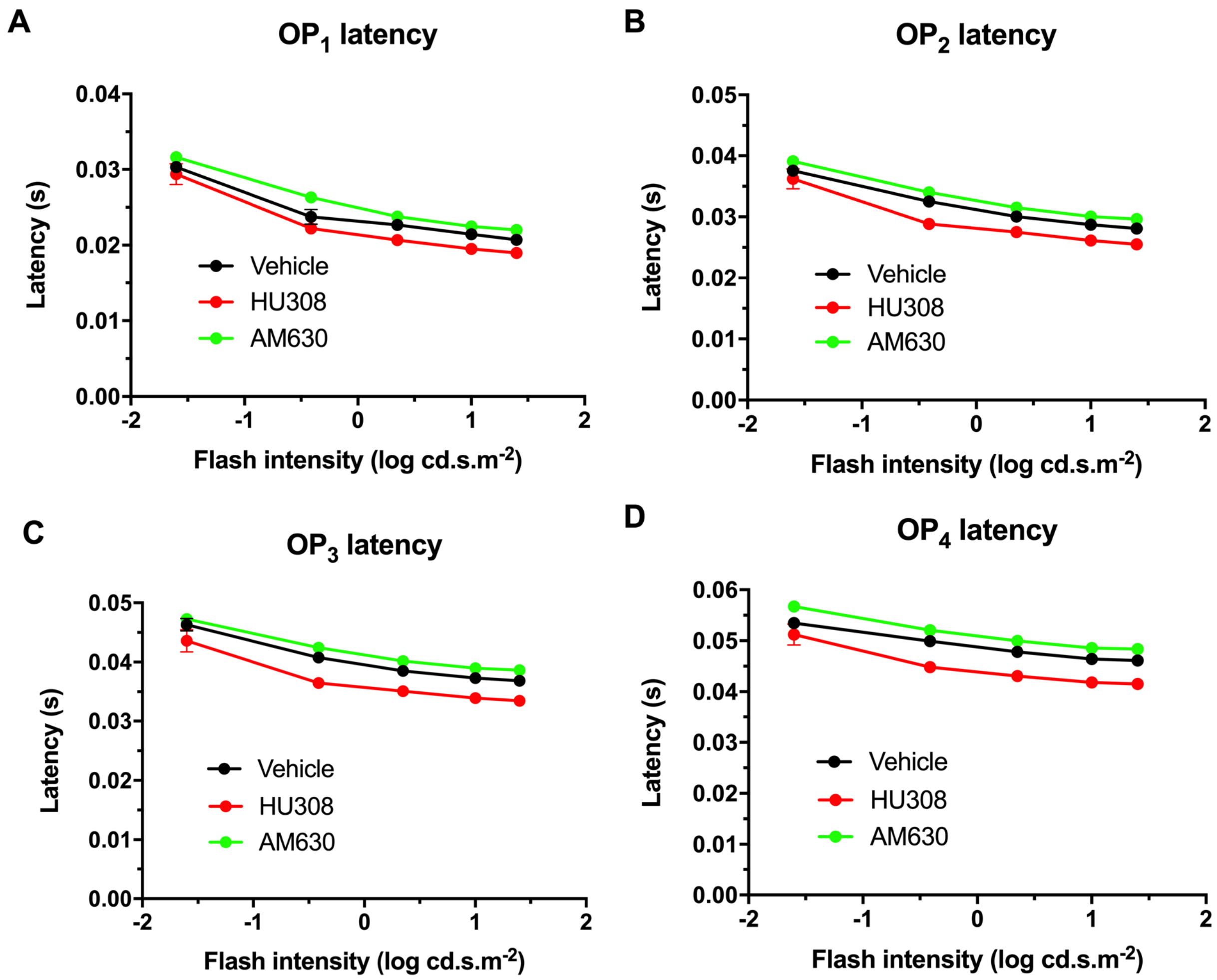

Supplementary Figure 3

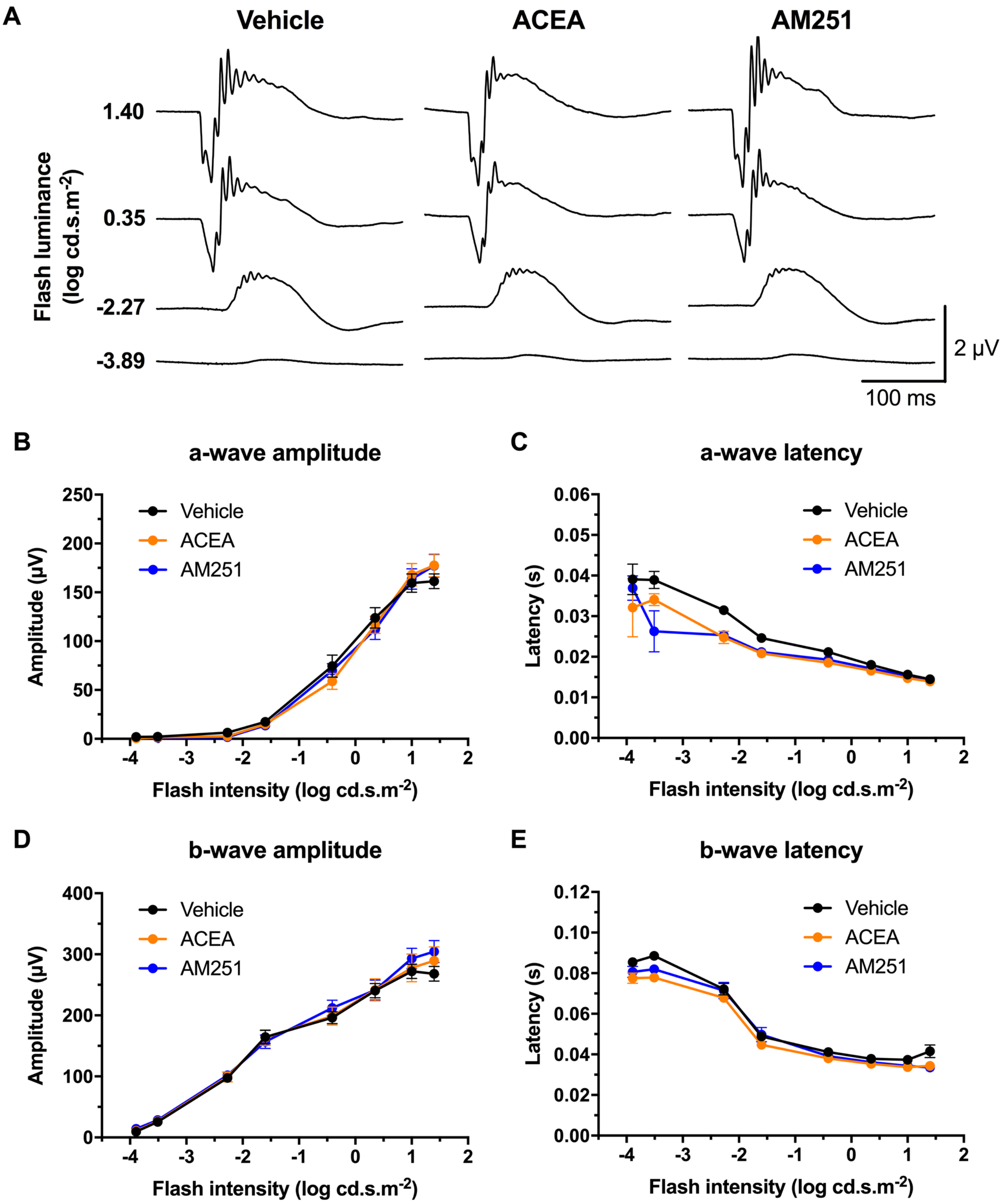

Supplementary Figure 4

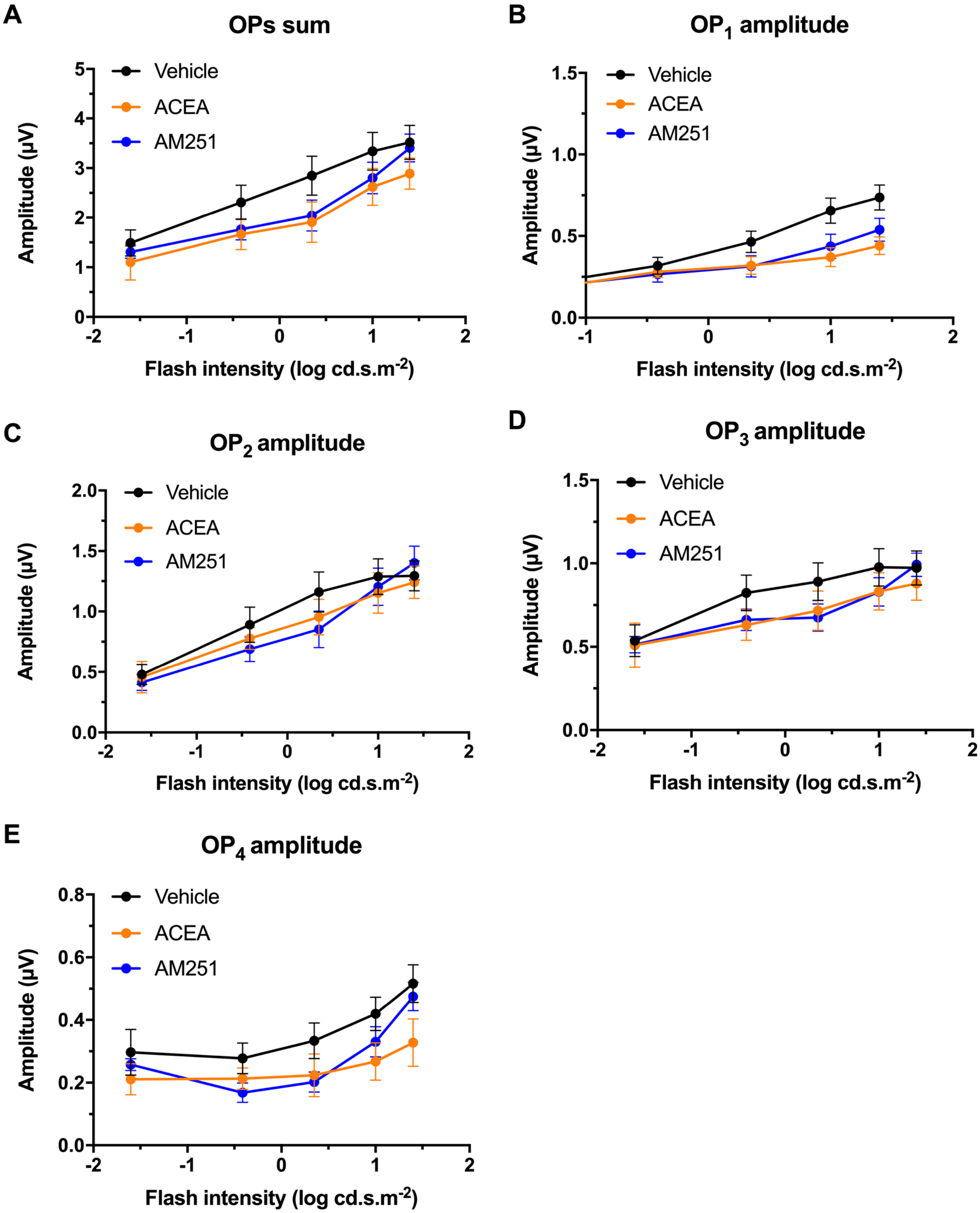

Supplementary Figure 5

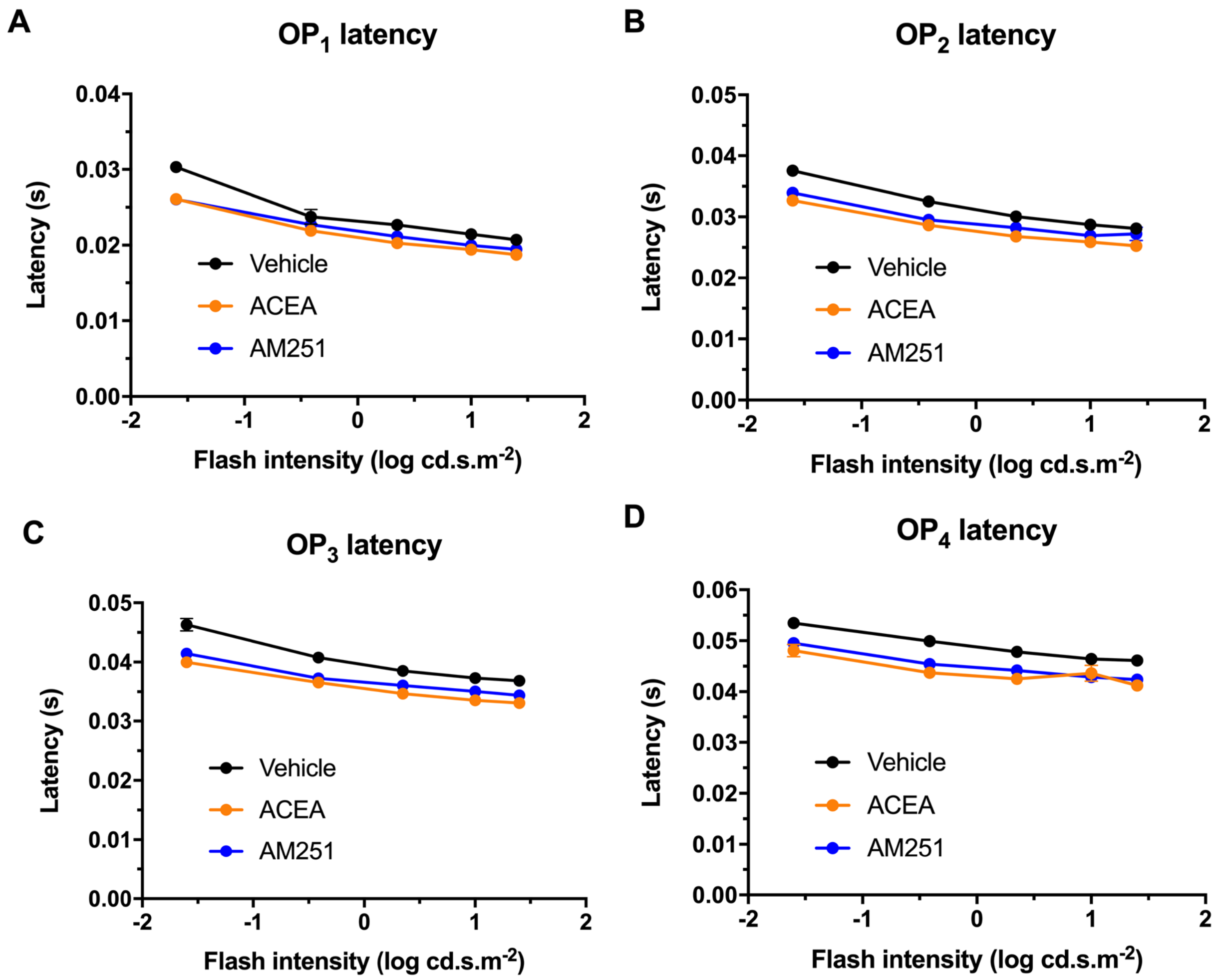

Supplementary Figure 6

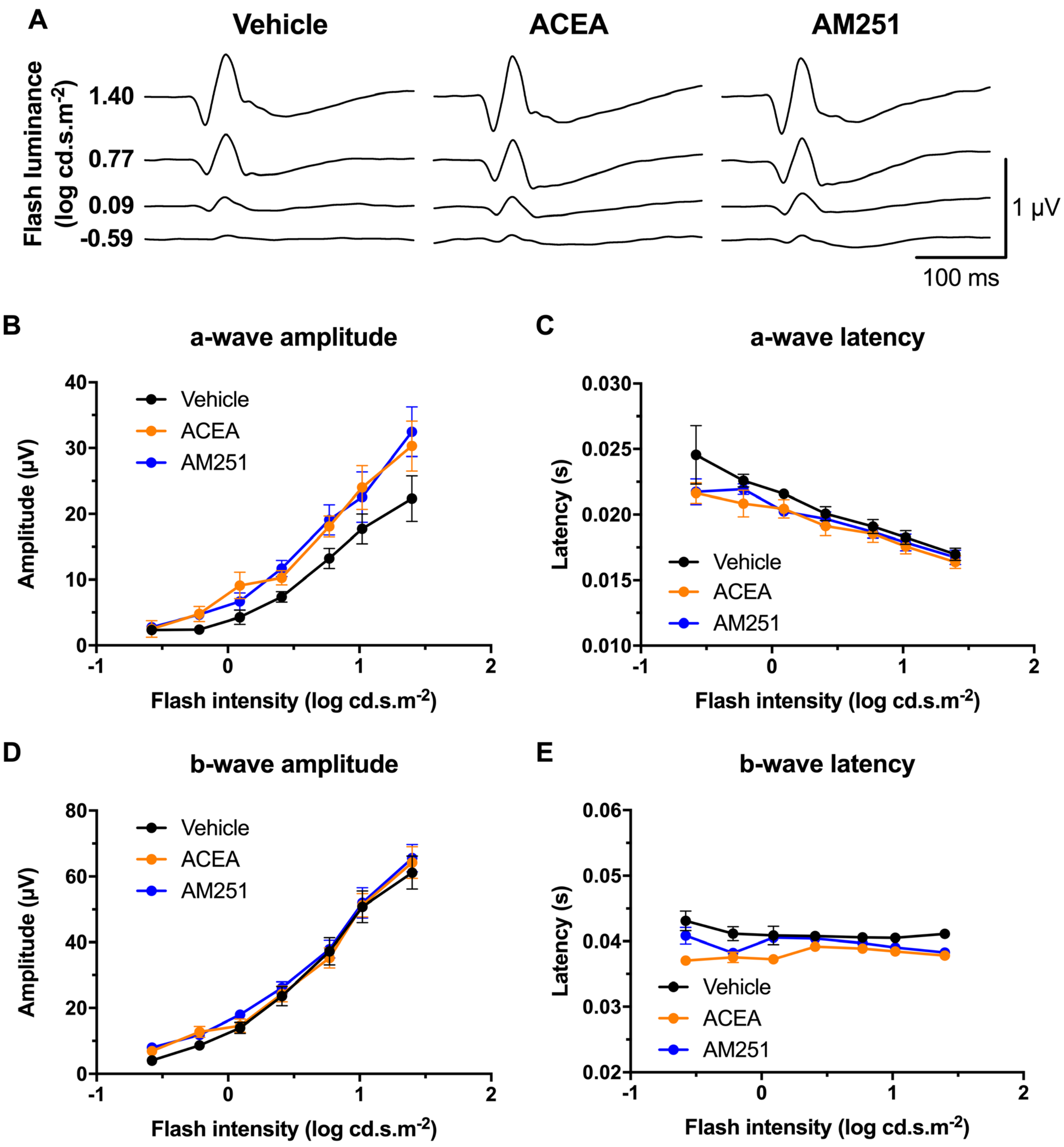

Supplementary Figure 7

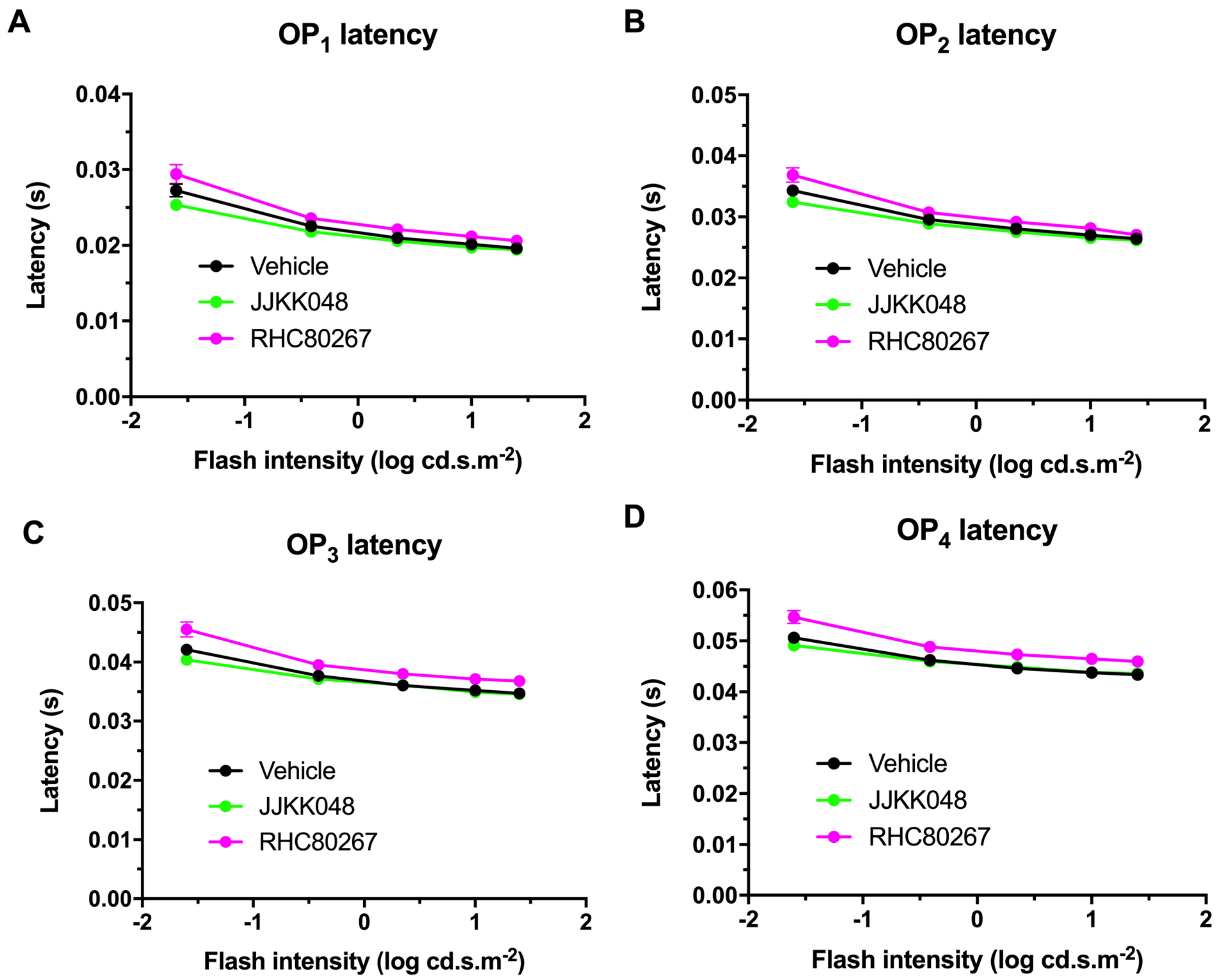

Supplement: Supplementary file 1 — Supplementary Information. [file 41598_2020_72553_MOESM1_ESM.pdf]
